# Genital Injuries: Are They Telling us Something about Sexual Violence?

**DOI:** 10.1055/s-0040-1701465

**Published:** 2020-02

**Authors:** Carolina Orellana-Campos

**Affiliations:** 1 Pediatric and Adolescent Gynecology Unit, Roberto del Río Children’s Hospital, Santiago, Chile; 2 Pediatric and Adolescent Gynecology Unit, Santa María Clinic, Santiago, Chile

**Keywords:** sexual assault, rape, genital injury

## Abstract

Genital injury has a forensic relevance after a sexual assault and it has been discussed and investigated among professionals who work in this field. To analyze the studies published in the last decades, the present review examines different factors that may influence this finding, first clarifying terms of the forensic field, such as the peculiarity of the legal medical examination, and the distinction of the terms “legal” and “anatomical” vagina. Finally, it analyses if it is possible that the existence of these injuries in victims explain the lack of consent in sexual contact, and to clarify the meaning of the absence of injuries.

## Introduction

In the usual context of medical practice, the clinicians assess, diagnose, document and treat body injuries according to the medical needs of the patients. When a victim of sexual violence is evaluated in the forensic context, much of the effort is put on the genital examination, giving too much emphasis to the presence of the genital lesions as evidence to take a legal action. For this reason, the forensic importance of genital lesions after a sexual assault has been subject to discussion and research among professionals working in this field.

Albeit understanding the psychosocial context and consequences of sexual violence is crucial for a better management of the victims, the aims of the present manuscript is, on the one hand, to refer only to genital injuries in adult females as an evidence of sexual assault. On the other hand, the present review intends to clarify the research in this area, which is full of uncontrollable variables, and therefore, it is not possible to make reliable conclusions considering genital lesion as the only evidence, even when they may be present after a consented intercourse. Before analyzing this current research and delineating the importance of genital lesions, it seems pertinent to clarify some terms and characteristics of this forensic field.

## Forensic Sexual Examination

The victim of sexual abuse requires to be examined by a competent clinician with a comprehensive knowledge of his/her forensic and therapeutic role. Essential components of a sexual forensic examination (Table 1) must be described in a standardized medico-legal report, with objective terms, providing expert opinion in legal proceedings, but in a language readable by police and lay people.^1^

**Table 1:** Components of the forensic examination

**1. Informed consent**
**2. Medical and gynecological background**
**3. History of the aggression**
**4. General physical examination** • **Search and collection of biological material**
**5. Genito-anal examination** • **Inspection:** • **Naked eye** • **Colposcopy** • **With blue toluidine dye** • **Search and collection of biological material**
**6. Documentation of injuries and other findings** • **Interpret and report the findings**
**7. Chain of custody**
**8. Management:** • **Prophylactic treatment of sexually transmitted infections** • **Emergency contraception** • **Derive**

It is important consider the clinical context in which the clinical environment in which physical and genital examinations were performed, and the expertise of the clinician. It is clear that clinicians or nurses with different training levels will bring different competencies to the clinical forensic examination, and this will influence their findings. The venue can be a referral unit, center or any variant of this model care (e.g., emergency unit). Both examiner and place heterogeneities may explain the different outcomes that a sexual forensic examination can have.^2^

The forensic examination can be done with several techniques to watch the genital zone, some of them allow covering the skin and mucosa with different solutions. Some people only use naked-eye inspection; others use the magnification given by a colposcopy, adding or not toluidine dye.^3^ As result, the technique has a key influence on the frequency of found injuries, and therefore, it is necessary to be cautious on its interpretation. Colposcopy has been shown to be statistically superior to gross visualization alone,^4^ which may be increased with toluidine blue staining.^5^ However, the latter might be overestimating microscopic injuries caused by ordinary wiping, insertion of tampons, sport activity or other day-to-day personal routines.^6^ Indeed, one study^7^ confirmed the suspicion that minor vaginal conditions, which do not deserve to be classified as ‘lesions’, are fairly common in women under normal circumstances.

## Pubertal Changes of the Genitalia

The vaginal epithelium is characteristically thin in childhood, but after puberty, it begins to thicken in response to estrogen stimulation with progressive cellular proliferation and growth that results in the formation of intermediate and superficial layers of cells,^8^ which could make the vagina more resistant to friction. Although it could be deduced that children may get genital injuries easily, most sexually abused children will not have signs of genital or anal injury, especially when examined non-acutely.^9^ Moreover, the primary predictor of diagnostic findings was not the age, timing of the examination, or the history told by the adult, but the history reported by the child.^10^

## The legal frame

Prior to continuing, it is important to define a legal frame of sexual violence. Jewkes et al^11^ define sexual violence as “Any sexual act, attempt to obtain a sexual act, unwanted sexual comments or advances, or acts to traffic, or otherwise directed, against a person's sexuality using coercion, by any person regardless of their relationship to the victim, in any setting, including but not limited to home and work” (p. 140). Governments in the Latin America and the Caribbean (LAC) region began to revise national legislations to address violence against women in the 1990s. Many countries incorporated specialized legislation based on a gender perspective, and reformed their civil and criminal codes accordingly. Many advances were linked to implementing international agreements at the national level.^12^ Besides the adoption of legislation, most countries of the region have formulated plans and programs oriented toward the prevention and eradication of violence against women, boys, girls and adolescents. Moreover, in some countries, legislative reforms have also addressed sexual harassment, sexual exploitation or violence in conflict settings.^13^

In many cases, legal reforms have reframed sexual violence as a criminal rather than a moral offense - as it was historically conceptualized in many LAC legal systems. In some settings, discriminatory clauses against the victims have been eliminated, such as allowing victims to be questioned about their previous sexual history, their conduct during the attack or their “honour.” Also, legal reforms have introduced marital rape as a criminal offense, which did not exist before in some countries, such as Mexico. For instance, in Brazil in 1983, Maria da Penha Maia, who was a woman who survived to two murder attempts by her husband. She became a paraplegic as a result of the abuse. She battled for twenty years to bring her case to justice, appealing to international organizations such as the Inter American Commission on Human Rights. The story gained international attention and finally the national domestic violence law in Brazil was signed in 2006 and named “Maria da Penha” in her recognition. The law specifically defines sexual violence as a crime, and includes preventive, punitive and protective legal mechanisms. It is considered one of the most advanced laws in the world addressing violence against women.^12^

Despite significant improvements to the laws addressing sexual violence in LAC, sexual violence still remains as a major concern. While in some countries marital rape is not addressed by the legal code and others still consider rape and sexual assault as an offense against “morals” or honor rather than a criminal act against the individual woman,^13^ in most developed countries the argument is the lack of consent.

Otherwise, it is quite interesting the difference between medical and legal definitions of vagina in some countries, as the UK, which may guide to confusion and error amongst professionals involved in rape allegation. The medical definition considers the vagina as a muscular tube that has the cervix as its proximal end and the hymen (or hymenal remnants) as the distal end. The British legal definition according to the Sexual Offenses Act 2003 (point 9, section 79, part 1) considers the distal end of the vagina as the beginning part of the vulva, therefore, “vagina” includes the vulva (between the labia). For legal purposes, penetration of the vagina does not have to involve penetration of the hymen. By contrast, in other nations there is no difference between both meanings in their domestic law.^14^

## Evidence about Genital Injuries in Sexual Violence

The wide variations found in the literature are attributable to diverse examination variables such as inconsistent definitions of “findings,” variable time span from sexual intercourse to examination, inclusion criteria of complainants, and divergent statistical methods. These variations demonstrate the difficulty of interpreting the findings in a group of victims, and it is even harder to try to compare them.^15^ Therefore, I will focus the analysis on genital and/or anal injuries only.

The prevalence of genital injuries reported after sexual assault ranges between 5 and 87%, according to a meta-analysis conducted by Kennedy.^2^ The same study found a mean prevalence of 34.8%. However, the authors claimed that they were unable to draw firm conclusions about the precise prevalence of genital injuries due to the heterogeneity of research methodologies. In a more recent and bigger work,^16^ genital injuries were detected in 22.0% of women examined at a sexual assault referral center (SARC). Nevertheless, while genital injuries were found in 24.5% of women who alleged complete vaginal penetration, only 13.2% of women with suspected sexual assault but no clear type of penetration had similar findings.

To improve the accuracy of interpretation of physical findings, some years ago a pattern of genital injuries in female victims was defined, whose acronym is TEARS^17^: Tears (lacerations), Ecchymosis (bruises), Abrasions, Redness and Swelling. Another classification considers abrasions, bruises and wounds, which can be lacerations or incisions.^18^ Many studies, however, have excluded erythema, redness and swelling when calculated injury rates as they are more subjective. These studies tend to have lower injury rates than those using the TEARS system, making comparison difficult.^3^

The frequency and type of injury also vary according to the region of penetration.^16^ In vaginal penetration, laceration, abrasion and bruise were observed in 13.1%, 11% and 5.7% of women, respectively. In anal penetration, abrasion and bruise were similarly found (8.6%, 2.9%, respectively), but laceration was more frequent (21.3%). The commonest sites, with at least one injury in vaginal penetration, were the posterior fourchette (7.4%), the fossa navicularis (6.8%), the labia minora (6.1%) and the hymen/hymenal remnant (6.0%). When penetration was anal, the frequent sites were the perianal (19%), the anus (9.8%) and the rectum (2.9%).

Another issue to consider is the timing of sexual assault and healing. The knowledge of injury healing may assist to decide the urgency with which an examination is performed. In some cases, the age of an injury might assist in determining who had access to the victim during the specified timeframe. Police or the court may ask the clinician to consider how old a genital injury is, and this may help to determine whether it was a result of previous consensual intercourse or a later alleged assault. On the other hand, many studies did not stipulate injury rates as genitalia tend to heal quickly.^3^

In terms of healing times, the evidence has shown that non-hymenal genital injuries heal at diverse rates depending on the type, location and severity. Nonetheless, there is no statistical difference in the rate of healing between pre- and postpubertal girls. In case of laceration, its depth determined the time required to heal. While superficial vestibular lacerations seemed to heal in 2 days, deep perineal lacerations required up to 20 days.^19^

The timing of examination determines the diagnostic rates of genital injuries,^20^ and women examined ≤ 72 hours after an assault have significantly more injuries than those examined > 72 hours. Anderson et al^21^ studied the changes in the pattern of genital injuries according to type, site, area and number at 48 and 72 hours of consensual vaginal intercourse using several techniques. In those women examined at 24 hours, they found a significantly higher number of injuries, and bigger surface areas of injury, both total and in the posterior fourchette, and bigger surface areas of abrasions and redness. Similarly, Zilkens et al^16^ found that the odds of observing a genital injury decreased with a delayed examination. Considering the techniques, the median survival time for lesions was 24, 40 and 80 hours using the naked eye, colposcope and toluidine blue dye, respectively.^22^

From a therapeutic and forensic perspective, a differential diagnosis is crucial. In both settings, the professional could be asked whether the genital findings resulted from an alleged assault or have another explanation. Therefore, awareness of medical conditions that affect the genitals can significantly reduce stress in patients and their surroundings, and lead to an accurate diagnosis. There are several conditions that might be confused with injuries such as allergy, eczema, psoriasis, infections (e.g., candida), and normal anatomical variations, amongst others. Consequently, obtaining a full history, when indicated, is a critical element to establish the context in which these findings should be interpreted.^3^ ^23^

## How to Interpret Genital Injuries when the Consent of the Victim is Questioned?

### Knocking Down Myths

Decades ago, there was a wrong view about the “normal” response of women in consensual intercourse. This included vaginal lengthening, increased lubrication and changes in muscular tension, which protected her from genital injury.^24^ By contrast, the use of force and the absence of this “normal physiological process” during sexual assault would make injuries inevitable. As result, a female genital injury would be treated not only as evidence of sexual contact, but also as lack of consent.^25^ ^26^ Indeed, this hypothesis is still in the popular imagination in some countries and their justice systems.

Another myth that must be tackled is that rapes or sexual assaults are necessarily violent. Modern legal definitions have now replaced the ‘use of force’ with ‘lack of consent’ as the defining feature of rape. In the Sexual Offenses Act 2003, a person consents if he/she agrees by choice, and has the freedom and capacity to make that choice,^27^ which requires active participation. This has changed how the crime of rape is conceptualized and prosecuted legally, and societies are also assimilating the basic awareness that the use of force is not a prerequisite for unconsented intercourse, and that physical resistance is not a universal response of the victims.^6^

Specifically about resistance, many women feel paralyzed when are attacked, especially when they fear for their life.^28^ In addition, it is frequent that women who did not respond aggressively blame themselves, and therefore, are less willing to talk about their experiences with others. Although a Danish study^29^ found that some women who resisted verbally during their assault were more likely to suffer a physical injury, no correlation was found between physical resistance and the risk of sustaining an anogenital injury. However, the lack of information about the time from assault to examination, and the differentiation between genital and anal injuries makes difficult to draw a firm conclusion from it.

The likelihood of injury in the first intercourse of a woman deserves a special mention. During many years, the bleeding from the first hymenal laceration has had sociological/religious significances in many cultures across the world. Nevertheless, hymenal injury is not always present, as 40 to 80% of women do not bleed in the initial coitus,^6^ and the hymen was observed intact in 52% of adolescents who admitted past intercourse.^30^ Indeed, when pregnant adolescents were examined for sexual abuse, 82% of the examinations were normal and only 7% were definitive for penetrating trauma.^31^ Hence, the likelihood of sustaining a genital injury is not related to the consent, resistance or prior sexual experience.

### Was the Sexual Contact Consented?

This is the most crucial question, which is not always answered ‘beyond reasonable doubt’. This situation usually frustrates prosecutors of sexual crime as it might explain low conviction rates for rape.^6^ The prevalence and location of genital injuries provide only a partial description of the nature of genital trauma, and the use of refined strategies of injury measurements has not assured this nature.^32^ For this reason, it is necessary to analyze studies where complainants of rape were compared with a control group, that is, consensual intercourse.

## Systematic Review of Case-control Studies of Genital Lesions

All case-control studies published until 31st December 2018 in PubMed, CINAHL and EMBASE databases were retrieved using the terms *case-control* OR *case* AND “*control*” OR “*consensual*” AND “*nonconsensual*” AND *genital* OR *anal* OR *genitoanal* OR *vagina* OR *vulva* OR *vulvar* AND *lesion* OR *injury* AND *sexual abuse* OR *sexual violence* OR *rape* OR *sexual assault*. The search retrieved 49 different articles, 41 of them were excluded after title and abstract reading, and full-text reading was performed for the remaining 8 articles, independently of the language. Finally, 6 articles were included considering the following inclusion criteria^1^: case-control studies,^2^ the primary outcome was the analysis of female genital lesions,^3^ cases were women who underwent sexual violence and controls were women who consented to sexual intercourse.

Table 2 analyses three of the most recent studies in which victims of a sexual assault were compared with a control group. McLean et al^33^ have found that the presence of injuries was significantly greater in the victims. However, the average time for the exam was significantly longer for the control group, and the only factor that showed an increased risk of injury was the relationship with the attacker, specifically with a close one. Astrup et al^34^ also found that the frequency of injuries was significantly higher in the victims, who have injuries in different places, are larger and more complex ones in comparison to the control group. However, the groups are small to generalize. Finally, Lincoln et al^35^ also found that the frequency of injuries was significantly higher in the victims, and the abrasions and bruises were observed exclusively in the cases. However, the average time from sexual penetration to examination was longer in the consensual group, even higher than showed by MacLean et al.^33^ Since genital lesions heal quickly, these conclusions may be questionable. In a retrospective research, Jones et al^36^ defined clearly that the presence of anogenital trauma suggests that penetration has occurred and that nothing tells about consent. In addition, anogenital injury is not an inevitable consequence of sexual assault – the lack of genital injury does not imply consent by the victim or lack of penetration by the assailant. But their results are biased as 15% of the nonconsensual group had had consensual intercourse within 72 hours of the reported assault. It is possible that anogenital injuries attributed to the sexual assault were actually secondary to prior consensual intercourse.

**Table 2:** Prevalence, pattern and severity of genital lesions in case-control studies

Author	Presence of injuries (No. Patients [%])		Affected area (No. of injuries [%])			Type of injury (No. of injuries [%])			Observations
	Cases	Controls		Cases	Controls		Cases	Controls	
**MacLean et al** 33 **(*n* = 500 cases / 68 controls)**	114 (22.8)*	4 (5.9%)	PF/FN Labia Vagina Urethra Hymen Cervix	69 (13.8) 38 (7.6) 11 (2.2) 10 (2.0) 9 (1.8) 4 (0.8)	3 (4.4) 1 (1.5) 0 0 1 (1.5) 1 (1.5)	Lacerations Abrasions Bruises	52 (10) 48 (10) 34 (7)	1 (2) 1 (2) 3 (4)	-Latency† within 48 hours -Average time of the exam was greater* for control group
**Astrup et al** 34 **(*n* = 39 cases / 98 controls)**	NE 10 (26%)* C 13 (33%) TB 13 (33%)	NE 4 (4%) C 9 (9%) TB 20 (20%)	OD PF/FN Labia Vestibule Clitoris Hymen C PF/FN Labia Vestibule Clitoris Hymen TB PF/NF Lips Vestibule Clitoris Hymen	NE 6 (43%) 4 (29%) 2 (14%) 2 (14%) 1 (7%) 11 (58%) 10 (53%)* 3 (16%) 2 (11%) 1 (5%) 11 (55%) 15 (75%)* 4 (20%) 2 (10%) 1 (5%)	NE 29 (85%)* 3 (9%) 1 (3%) 2 (6%) 1 (3%) 36 (75%) 11 (23%) 3 (6%) 2 (4%) 1 (2%) 42 (81%)* 16 (31%) 4 (8%) 2 (4%) 1 (2%)	NE Lacerations Abrasions Bruises Other C Lacerations Abrasions Bruises Other TB Lacerations Abrasions Bruises Other	11 (28%) 5 (13%)* 3 (8%) 14 (36%) 14 (36%) 6 (15%)* 4 (10) 19 (49%) 15 (38%) 6 (15%) 20 (51%)	31 (31%) 2 (2%) 2 (2%) 34 (24%) 41 (42%) 5 (5%) 3 (3%) 48 (49%) 49 (50%) 7 (7%) 52 (52%)	-Latency within 48 hours
**Lincoln et al** 35 **(*n* = 41 cases / 81 controls)**	22 (53.7%)*	8 (9.9%)	PF/FN Labia Vestibule Clitoris Hymen Vagina Cervix	15 (36.6%)* 12 (29.3%)* 3 (7.3%) 1 (2.4%) 4 (9.8%) 2 (4.9%) 2 (4.9%)	6 (7.4%) 0 (0) 1 (1,2%) 0 (0) 0 (0) 0 (0) 0 (0)	Lacerations Abrasions Bruises	13 (32%) 8 (20%) 9 (24%)		- Latency within 72 hours - Average time of the exam was greater* for control group
**Jones et al** 36 **(*n* = 204 cases / 51 controls)**	173 (85%)	37 (73%)	Hymen FN PF Labia Vagina	≈ 38% ≈ 50% ≈ 30% ≈ 35% ≈ 15%	≈ 59% ≈ 40% ≈ 25% ≈ 14% ≈ 10%	Lacerations Erythema Abrasions Ecchymosis Edema	≈ 40% ≈ 16% ≈ 25% ≈ 13% ≈ 5%	≈ 39% ≈ 30% ≈ 20% ≈ 8% ≈ 4%	- Retrospective - Small number of controls. - More than one type of assault was documented in 45% cases and 49% controls - Cases had recent consented intercourse

Abbreviations: C, examination by colposcopy; EG, external genital; FN, fossa navicularis; ma, majora; mi, minora; NE, examination with naked eye; PF, Posterior fourchette; TB, examination with toluidine blue.

* - Statistically significant difference.

† - Latency refers to the time elapsed between the sexual act and the physical examination.

Despite the works of Anderson et al^37^ and Kongtanajaruanun et al^38^ having been case-control studies, the way in which the results were delivered is too heterogeneous, and therefore, they cannot be added to Table 2. The former gave results on the pattern of injuries according to the number of sites and areas affected instead of the number of patients. Although the latter research provided information on the location and pattern of lesions, the latency of the examination was too broad to compare its results with those previously analyzed. In addition, it did not make clear reference to the total number of patients affected in each group.

## Factors Associated to Genital Injuries

Researchers have been motivated to determine whether factors associated with genital injury can assist in obtaining evidence, to corroborate something that typically occurs between two people without direct witnesses.

### Pattern or Severity of Genital Injury

Based upon the theory that genital injuries might be more likely to occur or be more severe in those cases without consent, the presence, pattern and/or severity of genital injury might be helpful in answering the question about consent. However, this hypothesis is outdated. Although the aforementioned studies done by Astrup et al^34^ and by Lincoln et al^35^ reported that cases had significantly more abrasions and bruises, and a higher frequency of multiple lesions, the small sample size of the former and the delayed cases examination of the latter affect this presumption. Anderson et al^37^ shows that there were differences in the types of injuries and the total numbers of injuries between the nonconsensual and the consensual groups. Except for redness, there were more sites of injury in the nonconsensual group than in the consensual group, where lacerations, ecchymosis, and abrasions were greater in the nonconsensual than in the consensual group. One remaining chance to keep this hypothesis as partially valid is the use of standardized scales such as the Genital Injury Severity Scale developed by Kelly et al^15^ to define and measure external genital injury after sexual intercourse. However, they need to be validated prospectively in an unbiased/unselected population.

### Location of Genital Injury

Astrup et al^34^ described that victims had a higher frequency of lesions in locations other than the 6 o'clock position. However, controls had a significantly higher frequency of lesions in the 6 o'clock position than cases when the naked eye and toluidine blue dye were used. Also, the cases had a significantly higher frequency of lesions on the labia than controls when colposcope and toluidine blue dye were used. None of the investigated women had lesions in the vagina or cervix.

According to Lincoln et al,^35^ the fossa navicularis was the most common genital site for an injury seen overall. In the consensual group, injuries were seen at only four sites: the posterior fourchette, the fossa navicularis, the perineum and in the periurethral area. In the nonconsensual group, injuries were seen at 10 sites with the fossa navicularis and the labia minora the most frequently injured, and both were statistically more affected in the nonconsensual group. Also, while injuries at the posterior fourchette were seen in both groups, this site was statistically more affected in the nonconsensual group.

### General Body Injury

Taking into consideration the relative irrelevance of common types of genital injury, nongenital examination and documentation of injuries elsewhere on the body may be invaluable. In the meta-analysis published in 2013^2^ about injury data in sexual violence, the mean prevalence of general body injury was 48.6%, with a range between 6.3 and 82%, and a median of 47.4% in complainants of sexual violence. Other studies^20^ ^33^ have shown that there is a higher chance of finding an injury on body surfaces other than the anogenital area. Zilkens et al^16^ studied a subgroup of 807 women with completed vaginal penetration who consented to both general body and genitoanal examinations, and 69.8% of them had general body injuries. They demonstrated that women with a general body injury were more likely to present a genital injury, although the risk was only 1.6-fold.

### Vaginal Penetration by Body Parts other than the Penis

Penetration with finger(s) and possible pre-existing genital ‘infection’ were found to be significantly associated with the presence of genital injury in the univariate analysis performed by Lincoln et al.^35^ Logistic regression demonstrated that a penetration that included finger(s) was 4.2-fold more likely to result in ≥ 1 genital injury than penetration without fingers. While the presence of lacerations was less likely than other injuries if penetration involved finger(s) or if a woman was penetrated exclusively with finger(s), abrasions were more likely to occur than other injury types in the same scenario. Similarly, Zilkens et al^16^ reported that genital injury was more likely with multiple types of penetrants (5.0-fold) other than the penis such as finger(s) or hand.

### Vaginal Penetration by an Object

Although in the Lincoln et al^35^ study there were no women exclusively penetrated with an object, five women gave a penetration history that included an object. Four of these 5 women were penetrated consensually and none of them sustained any injury; the nature of the object was not recorded in the consensual group. One woman was penetrated nonconsensually with an object, who described it as a ‘toilet-roll holder’ and was found to have three bruises on the labia minora and hymen. Zilkens et al^16^ also reported that genital injury was more likely with multiple types of penetrants, among them an object.

### Age of Victim

Hilden et al^39^ reported that age was significantly related to the occurrence of anogenital injury. Women ≤ 19 years-old and >50 years-old had the highest risk. Nevertheless, other studies^33^ ^34^ ^35^ showed that age is not an important factor for having genital injuries in sexual intercourse, either consensual or not.

### Previous Sexual Activity

When White et al^40^ compared the findings in virgin and non-virgin adolescents (12–17 years old) seen at a SARC after an allegation of nonconsensual intercourse, they did not find significant differences for the presence of genital or nongenital injuries overall. When different genital sites were taken into account, 50.6% of the participants from the virgin group had a hymen injury, but only 12.4% of adolescents from the nonvirgin group had it. Other sites were similarly affected. Nonetheless, the virgin adolescents consulted later than the nonvirgin group (90 versus 44 hours), which may affect the injury rate.

Recently, Zilkens et al^16^ found that 52.1% of virgin women who reported completed vaginal penetration had genital injury. This represented a 4.7-fold risk of genital injury if there was no history of prior vaginal intercourse, which was the highest factor. However, this research did not have control cases.

### Nearness of the Aggressor to the Victim

While Hilden et al^39^ found that assaults by strangers were less likely to cause anogenital injury, although this was not statistically significant, McLean et al^33^ demonstrated that if a woman knew her assailant, then there was a statistically significant higher change of sustaining an injury when compared with women who did not know their assailant. In the same way, Maguire et al^20^ found that to be a victim of sexual violence by an acquaintance increased the risk of genital injury 2.3-fold. Hence, it seems that the nearness of the victim to her attacker may be a factor to consider.

### Other Factors

Both the studies by Astrup et al^34^ and by Lincoln et al^35^ analyzed other factors such as time since intercourse, use of condoms/lubricants, insertion of a tampon in 72 hours preceding the examination, usual pattern of sexual activity, roughness of intercourse, and previous vaginal deliveries. None had any significant influence on the presence or type of lesions irrespective of the examination technique. Concerning alcohol consumption and sedatives by the victim previous to the assault, Hilden et al^39^ found that > 50% of the sexually assaulted women in their study were influenced by alcohol. When the amount of alcohol drunk was enough to induce amnesia, then the anogenital injury rate was lower. A lesser resistance imposed by the victim could explain this. Nonetheless, Maguire et al^20^ concluded that alcohol use had no effect on the frequency of genital injury. Also, Zilkens et al^16^ reported that the presence of a genital injury was less likely with sedative use. Genital injury was not found to be significantly associated with a previous history of vaginal delivery, obstetric genital injury, surgery, or if women had a pigmented skin.^33^ ^35^ Regarding the effects of hormonal contraception on the presence of genital injuries, the evidence is contradictory. While Lincoln et al^35^ did not detect a significant relationship between hormonal contraception and the presence of injury, another study^20^ that included the use of colposcopy found a significantly higher rate of genital injury in those women who were not taking hormonal contraception. Finally, it has been reported that females in dorsal decubitus with thighs flexed, and male/female genital disproportion, would be predisposing factors to vaginal injury in both nonconsensual and hurried consensual intercourse.^41^

## Lack of Injuries

The percentage of participants without body or genital injury in diverse studies was extremely wide, between 18 and 68%.^2^ This may be explained by several factors, some of them detailed above, which must be kept in mind. Therefore, the forensic clinician must collect all data, consider differential diagnoses, and then assess if the available evidence is compatible or not with the history provided by a complainant.^3^

It is known that police approach to the rape crime is significantly influenced by the presence of injuries. However, the examiner clinician must emphasize in his/her report that “the absence of genital trauma does not preclude the possibility of nonconsensual sexual intercourse.” As health care providers, we have an important obligation in ensuring that police officers fully understand the meaning and the significance of this assertion. Therefore, the same analysis that is discussed about genital injuries should be provided to the police, lawyers, the judiciary and the general public.^42^

## Discussion

Many factors influence the presence or absence of genital injuries during a sexual assault, and therefore, they are not strong evidence for this type of crime per se, but the existence of a mutual consent among two – or more – people involved in an intercourse. Although two serious studies demonstrated a significant difference between consented and nonconsented intercourse groups in prevalence of genital injury, they have serious methodological issues that may be affecting these results. Therefore, the presence or absence of genital injury should not be used to render an opinion regarding consent to sexual intercourse.

There are many reports in the literature concerning the prevalence of genital injury following alleged sexual assault. Unfortunately, the variety of examination/visualization techniques, participant inclusion criteria, injury definitions, and time to examination seen in these studies make them difficult to assess and get to a convincing conclusion.^16^ Thus, it is crucial to standardize the means used for detecting genital injuries, and to agree on injury definitions and examination protocols. The macroscopic genital examination may be the only reliable way to detect differences in injury typology and pattern if they exist.^35^ Also, it must be emphasized the importance of the time from sexual assault to examination as it is used in trials as well.^21^

Regarding the severity or pattern of genital injuries and their association with the consent, some skepticism must be applied to all research that tries to prove this association.^25^ Nevertheless, as a couple of studies reported that certain type of injuries and their location were seen exclusively in the nonconsensual group, further research is necessary to confirm this. On the other hand, the presence of lesions in other parts of the body may be of greater importance than minor genital injuries. Hence, this reinforces the idea that a complete physical examination is needed when we care for a victim of sexual violence.

Finally, the lack of injuries highlights the importance of ensuring that all those who are involved in these cases, such as medical and legal professionals, police officers, and the public in general, are aware that injury is absolutely not a necessary outcome of sexual violence.^2^

## Conclusion

In conclusion, it is important to emphasize that the absence of genital lesions does not translate the absence of sexual violence. This is something that we, as healthcare professionals, must be sensitive to and aware of, especially knowing the reality of the legal framework in our LAC region. Therefore, the forensic examination, although relevant, must be accompanied by other social, psychological, medical, etc. evaluations and care by the corresponding professionals to give evidence of the occurrence of a sexual assault as well as to give a comprehensive, holistic management to the victim.
